# Understanding the antepartum depressive symptoms and its risk factors among the pregnant women visiting public health facilities of Nepal

**DOI:** 10.1371/journal.pone.0214992

**Published:** 2019-04-04

**Authors:** Deepak Joshi, Santoshi Shrestha, Nipun Shrestha

**Affiliations:** 1 Health and Nutrition Department, Save the Children, Kathmandu, Nepal; 2 Global Health Alliance Nepal, Kathmandu, Nepal; 3 Institute for Health and Sport, Victoria University, Melbourne, Australia; USC Keck School of Medicine, Institute for Global Health, UNITED STATES

## Abstract

**Introduction:**

Antepartum depression is a contributing factor for adverse maternal and perinatal outcome. The study aimed to assess the antepartum depressive symptoms in selected public health facilities of Nepal.

**Methodology:**

This is a mixed-method cross-sectional study that included 143 pregnant women attending the antenatal checkup in four public health facilities of Kathmandu. Edinburgh Postnatal Depression Scale (EPDS) tool with cut-off score > = 10 was used to assess the antepartum depressive symptoms. Bivariate and multivariable analysis was carried out to identify factors associated with the depressive symptoms. Further semi-structured interviews were conducted with 12 pregnant women identified with the depressive symptoms.

**Result:**

Of the total 143 pregnant women, 26 (18%, CI at 95% 12.6–25.5) reported depressive symptoms. Multivariable analysis reported higher odds of antepartum depressive-symptoms with health problem, early gestational age, sex preference, and spousal alcohol intake. Thematic analysis of qualitative data further revealed participants’ apprehension on; birth outcome, a family expectation of male child, inadequate support from the family/husband and disturbed family environment.

**Conclusion:**

Notable proportion of pregnant women were reported with antepartum depressive symptoms. Women’s perception on patriarchal values for childbirth was revealed to be important factor for the depressive symptoms. The study draws an attention to a need for screening for antepartum depression into primary health care system. Strengthening ongoing efforts on gender equity could contribute the psychological well-being of pregnant women.

## Introduction

Antepartum depression can lead to adverse perinatal outcome like low birth weight, premature delivery, growth retardation, disrupted cardiorespiratory regulation, diarrhea and poor feeding [1–5]. Furthermore, the depression also attributes to occurrence of preeclampsia [6] and postpartum depression in women [6–8]. A review study has reported the prevalence of antepartum depression to be 15.6% in low and middle-income countries (LAMIC) [9]. Predictors determining perinatal depression, also includes antepartum depression, and child health outcomes are more common in LAMIC than in high income countries [10].

In Nepal, there are few published studies reporting the antepartum depression [11,12]. A recent study to assess common mental disorders among pregnant women in earthquake affected districts of Nepal pointed to an alarming prevalence (39%) of common mental disorders during pregnancy [12]. Although, the findings of this study do not apply to normal setting, the study does direct towards the relevance of antepartum depression as a scope of study in Nepal.

Despite Sustainable Development Goal prioritizes the promotion of mental health [13], this is still a neglected component of primary health care in Nepal. Given this context, antepartum depression also barely stands a chance to be in a public health priority in Nepal. Under the routine government healthcare service, health professionals providing antenatal services care are not trained to deliver mental health services. Hence the diagnosis of antepartum depression is frequently missed and has serious repercussions for maternal and child health. Furthermore, the literature has suggested that rates of depressive symptoms during pregnancy is not well known in Nepal due to a lack of a wide scale study.

This study aims to identify the risk factors associated with antepartum depressive symptoms in women visiting the public health facilities. Edinburgh Postnatal Depression Scale (EPDS) tool has been employed to assess the depressive symptoms. Studies across different countries have used EPDS tool to assess depression during pregnancy [12,14–17]. Though the scale is not a measure of clinical diagnosis, it does reflect the possibility of depression.

## Methods

### Study design and participants

This was a mixed method cross-sectional study that was carried out in four peripheral public health facilities; three primary health care centers (PHCCs) and one health post (HP) of Kathmandu valley namely; Tokhachandeswori PHCC, Ramkot PHCC, Nayapati PHCC and Budhanilkantha Health Post. Both PHCC and HP are peripheral level public health facilities providing basic health care services. PHCCs are led by medical doctors and they each cover an electorate constituency. HPs, on the other hand, are led by paramedics and have smaller coverage area called *ilaka*. In the catchment area, the female literacy rate had been reported to be 70% and the total population of women of reproductive age group (15–49 years) was 11,096 [18].

We estimated a total sample size of 143 pregnant women assuming a proportion of 24% with a confidence level of 95% and margin of error at 7%.

Two public health students in their final year of graduation and one nursing graduate received 3 days of training including one day field trial. All the trained field researchers were female. Data collection was carried out in June 2017, and paper-based tools were used for recording the information. Field researchers had no prior established relationship with the participants. They coordinated with the health workers of the study site and in accordance to their suggestion, made visit-plan. During the visit, they approached all pregnant women attending ANC at the health facilities until they obtained the sample size. There were 2 research tools; structured and semi structured questionnaires, that were administered through face to face individual interview. Structured questionnaire including the EPDS was administered with all 143 pregnant women after they received antenatal care service. Prior to the interview, consent was read to all the women, and an informed consent was obtained from those agreeing to participate in the study. One of the women did not consent to the interview. Each interview lasted for 35–50 minutes.

Further semi-structured interviews were conducted with the participants identified with the depressive symptoms to understand their perception and risk factors. There were altogether 26 pregnant women with depressive symptoms (EPDS cut-off score > = 10) who were approached for further interview, however only 12 of them consented. Characteristics of these 12 participants are presented in Table 1. Other 14 participants, who did not consent, were from 18–33 years age group, EPDS score of 10–17 and from all pregnancy trimesters. Further reasons for non-participation were not recorded. During the interviews, only one field researcher was present with participant. These interviews were conducted in a convenient location around the clinic agreed by the participants. Interviews lasted for 30–50 minutes.

**Table 1:** Characteristics of the participants from semi structured interview.

Participant	Age (years)	Education	Living children	Gestational age (months)	EPDS score
1	22	Higher secondary	No	9	11
2	24	Lower secondary	1 Son and 2 daughters	8	12
3	30	Read and write	1 Son and 2 daughters	7	12
4	30	Lower secondary	1 Son	9	11
5	23	Secondary	1 Son	8	12
6	25	Intermediate	No	8	13
7	23	Primary education	1 Son	9	11
8	20	Higher Secondary	1 Son and 1 daughter	1	15
9	29	Lower secondary	No	8	14
10	18	Lower secondary	No	4	12
11	25	Read and write	No	7	15
12	22	Secondary	No	1	16

### Measurement of depression

EPDS was used to measure the depressive symptoms among pregnant women. This scale is primarily used for measuring the depression during the postnatal period; however, studies have suggested that the tool is reliable to screen for depression during the pregnancy [19,20]. Recommendation for cut-off score from several EPDS validation studies, carried out across the world, varied [21]. In Nepal, there are only few EPDS validation studies conducted for postpartum depression [22,23] but to our knowledge no validation study has been done for antepartum depression. A recently conducted study in Nepal used EPDS > = 10 cut-off score to report for common mental disorder [12]. Our study has also used an EPDS cut-off score of > = 10 that has been recommended for assessing both the minor and major depressive symptoms[15].

Participants who did not provide informed consent for the study, and had life threatening illness, were excluded from the study. This study assessed antepartum depression for all three trimesters of pregnancy [11,20,24]. Two public health professionals translated the tool into Nepali that was finalized after series of revisions from pretesting, field researchers training and field trial. All the respondents were instructed to answer the questions based on their last 7 days experience. Field researchers ensured that participants did not interact with any other individuals while answering the questions. The tool included 10 items with a rating scale from 0 to 3 thus the total score ranged from 0 to 30. Cronbach’s alpha was reported to be 0.72 for EPDS scale items in this study.

### Quantitative data analysis

Quantitative data were entered into MS excel 2013 and was exported into the IBM PASW version 21.0 for the analysis. Considering the small sample size, we recoded the explanatory variables to concise their categories. Chi-square test was performed for bivariate analysis, and variables whose p-value was found below 0.2 were further entered into the multivariable logistic regression model to assess the independent effect of each independent variable. The dependent variable was dichotomized as 0 = *non-depressive symptoms* and 1 = *depressive symptoms*. Multicollinearity test for the explanatory variables and goodness of fit was performed prior entering into the model. Odd’s ratio was used to present the findings measured at 95% CI.

### Qualitative data analysis

All the interviews were conducted in Nepali and noted in a diary. On the same day, field researchers clearly detailed and transcribed the notes in Nepali. Later, these transcripts were translated into English. Considering the time limitation, it was not possible to share the transcripts with the participants for their feedback. Thematic analysis was employed to process the data. Two coders (SS and DJ) were involved in the coding process. The analysis primarily followed inductive approach and identified the emerging codes, and to some extent, deductive approach was also used to identify the codes guided by the interview tool. Team manually performed the coding work, and finally decided four major themes from the interviews (Table 2). COREQ checklist was followed to ensure the quality of the content reported [25].

**Table 2:** Codes, sub themes and themes generated from the participants.

Themes	Sub-themes	Codes
Worried on birth outcome	Adverse effect due to health problem	Existing health problem of woman
		Existing health problem of husband
	Fear of miscarriage	Previous miscarriage
		Others experience of miscarriage
	Perception on complications	Small body size
		Others opinion on delivery
Sex preference	Family recognition	Husband’s desire for son
		family care and support
		Son as bread earner
	Social recognition	Earn respect and limelight
		Son as symbol of strength
	Fear of becoming pregnant again	birth of girl child
Inadequate family care and support	Inadequate supplement	No nutritious food
		No companion for antenatal check up
		Husband’s time
	Lack of support	Household chores as before
		Managing study time
Disturbed family environment	Disturbed relationship	Conflict among family members
		Interaction among the family members
	Financial hardship	family income

### Ethics

The study obtained approval from Ethical Review Board at Nepal Health Research Council. Approval was also obtained from the health facilities prior to the implementation. Pregnant women visiting these health facilities for an antenatal checkup (ANC) were recruited in the study after obtaining their informed consent.

## Results

### Quantitative findings

#### Socio-demographic profile

A total of 143 pregnant women were interviewed. Table 3 shows the characteristics of the participants. Their mean age was 24 years (SD 4.1 years) and average age at first pregnancy was 21 years (SD 3.6 years). Majority of them (61%) were pregnant for the first time. By occupation, only one fourth (25%) of the participants were engaged in income-generating activities.

**Table 3:** Characteristics of 143 pregnant women participated in the study from selected health facilities of the Kathmandu.

Characteristics	Total
***Age in years (Mean ± SD)***	23.8 ± 4.1
***Age at first pregnancy in years (Mean ± SD)***	21.3 ± 3.6
***Duration of marriage in years (Mean ± SD)***	3.9 ± 3.7
	**N(%)**
***Education***	
No formal education	27(18.9)
Formal education	116(81.1)
***Occupation***	
Income generating	36(25.2)
Non income generating	107(74.8)
***Pregnancy Trimester***	
First	10 (7.0)
Second	54 (37.8)
Third	79 (55.2)
***Gravidity***^***a***^	
Primigravida	87(60.8)
Multigravida	56 (39.2)
***Health Problems***^***b***^	17 (11.9)
***Planned Pregnancy***^***c***^	91 (63.6)
***History of abortion***^***d***^	16 (11.2)
***Partner's education***	
No formal education	12 (8.4)
Formal education	131 (91.6)
***Partner's Occupation***	
Business/service	80 (55.9)
Labour/Daily wage	63(44.1)
***Partner's presence during the ANC period***	126 (88.1)
***Alcohol Intake by Partner***	29 (20.3)
***Sex preference*** ^***e***^	32 (22.4)
***Family Type***	
Nuclear	62 (43.4)
Joint/Extended	81 (56.6)
**Depressive-symptoms (> = 10 EPDS score)**	26 (18.2)

^a^ Gravidity is the number of times a woman gets pregnant; primigravida if pregnant for the first time and multigravida if pregnant for more than one time.

^b^ Health problems reported by respondents prior the pregnancy that includes; hypertension, diabetes, TB, renal calculi etc.

^c^ Planned pregnancy is the pregnancy that was planned before getting pregnant

^d^ History of abortion includes both the induced and spontaneous one

^e^ Sex preference is the preference of family for male child

#### Rate of depressive-symptoms

Of the total 143 respondents, 26 [point prevalence 18%, CI at 95% 12.6–25.5]) were reported with antepartum depressive-symptoms.

#### Risk factors associated with depressive-symptoms

Table 4 shows bivariate and multivariable analysis of the factors associated with depressive symptoms. Only factors with a p-value less than 0.2 in the bivariate analysis were included for multivariable analysis. These variables include; age at first pregnancy, duration of the marriage, gestational period, gravidity, health problems, planned pregnancy, history of abortion, alcohol intake by partner and sex preference. Pregnant women with health problems had higher odds (AOR 4.8, CI 1.1–20.7) of the depressive symptoms than their other counterparts. Likewise, pregnant women who reported sex preference for the child (AOR 3.7, CI 1.2–11.6) and their husbands took alcohol (AOR 3.7, CI 1.2–11.4) were more likely to report the depressive symptoms.

**Table 4:** Multivariable analysis showing the risk factors associated with the antepartum depressive-symptoms who visited for antenatal checkup in selected health facilities of Kathmandu.

	OR (95% CI)	p-value	aOR (95% CI)	p-value
**Age** ^*^	1.05 (0.95–1.16)	0.29		
**Age at first pregnancy** ^*^	**0.83 (0.70–0.980**	**0.032**	0.85(0.69–1.04)	0.11
**Duration of marriage** ^*^	**1.12 (1.01–1.25)**	**0.022**	1.03(0.85–1.26)	0.73
**Education**				
No formal education	1.37(0.49–3.8)	0.547		
Formal education	Reference	-		
**Occupation**				
Income generating	1.41(0.55–3.59)	0.47		
Non income generating	Reference	-		
**Gravidity** ^**a**^				
Primigravida	**0.32(0.13–0.78)**	**0.012**	0.65(0.11–3.9)	0.63
Multigravida	Reference	-	reference	
**Gestational period**				
First Trimester	**4.40(1.23–15.76)**	**0.023**	**9.9(1.9–51.9)**	**0.007**
Second/third trimester	Reference	-	reference	
**Prior health problems** ^**b**^	**5.33 (1.82–15.62)**	**0.002**	**4.8(1.1–20.7)**	**0.036**
**Unplanned Pregnancy** ^**c**^	2.0(0.84–4.72)	0.11	1.98(0.67–5.8)	0.21
**History of abortion** ^**d**^	**3.2 (1.04–9.82)**	**0.041**	2.5(0.5–12.9)	0.26
**Partner’s education**				
No formal education	1.56 (0.39–6.23)	0.525		
Formal education	Reference	-		
**Partners Occupation**				
Business/Service	1.61(0.066–3.9)	0.28		
Labour/Daily wage	Reference	-		
**Presence of husband**	0.68(0.20–2.30)	0.54		
**Alcohol intake by Partner**	**4.0(1.59–10.18)**	**0.003**	**3.7(1.2–11.4)**	**0.023**
**Smoking habit of partner**	1.78 (0.66–4.81)	0.251		
**Sex preference**^**e**^	**4.15(1.67–10.31)**	**0.002**	**3.7(1.2–11.6)**	**0.024**
**Family Type**				
Nuclear	0.63(0.26–1.55)	0.32		
Joint	Reference	-		

*OR shows greater risk per unit (one year)

^a^ Gravidity is the number of times a woman gets pregnant; primigravida if pregnant for the first time and multigravida if pregnant for more than one time.

^b^ Health problems reported by respondents prior the pregnancy that includes; hypertension, diabetes, TB, renal calculi etc.

^c^ Planned pregnancy is the pregnancy that was planned before getting pregnant

^d^ History of abortion includes both the induced and spontaneous one

^e^ Sex preference is the preference of family for male child

### Qualitative findings

Semi structured interviews were conducted to complement the quantitative finding. Themes emerged from the interviews were **worried on birth outcome** with subthemes *adverse effect due to health problem*, *fear of miscarriage* and *perception on complication*; **sex preference** with subthemes *family recognition*, *social recognition* and *fear of becoming pregnant again*; **inadequate care and support** with subthemes *inadequate supplement* and *lack of support*; and lastly **disturbed family environment** with subthemes *disturbed relationship* and *financial hardship* (Table 2).

#### Worried on birth outcome

Most of the participants were worried on birth outcome whether the baby would be alive or not, and if alive would she/he be in a good health condition. Some participants related their worry with earlier stressful life events like miscarriage, and they feared recurrence of such events. Discordant to qualitative finding, multivariable analysis could not show *history of abortion* associated with depressive symptoms in this study.

*“Last year I had miscarriage*, *I do not know what will happen to this one*.*” Participant 12*

Some participants were distressed about their health problems, either existing or previous one, potentially affecting their baby. Similar finding was also reported from quantitative data where health problem of the women was found to be significantly associated with the depressive symptoms.

*“My health problems started with pregnancy like discharge from uterus and infection in urinary tract*. *Although I have treated but haven’t fully recovered yet*, *so I am worried whether this will affect my baby*.*”* Participant 2

Some participants expressed that complications during pregnancy and delivery could adversely affect the health of mother and baby. Such perception was usually a result of their interaction with family members, neighbors and friends. A young participant elicited her anxiety recalling a recent experience;

*“My relatives and other people said that I am much younger to give birth*, *and that due to my small body size there will be more difficulties to give birth*. *They say there may be a complication due to this*. *I often think if I won’t be able to give birth or if something bad happens to me or to my baby during the delivery*. *These feelings upset me often*.*” Participant 10*

One of the participants shared her fear and distress of undergoing surgical procedure during delivery.

*“I want to have normal delivery that is why I come for routine checkup*. *I even fear to think about the operative procedure during the delivery*. *I wish my worries will disappear*.*”* Participant 5

#### Sex preference

Confirming the quantitative result, sex preference was another key factor revealed from the qualitative data. Participants reported that their family including husband wished for a male child because son represents family lineage. Some of the participants mentioned that even they wished for a son who is a symbol of prestige in a family and society. Such belief simultaneously attributed to both pressure and joy.

*“This pregnancy could change my life*. *If there will be a son*, *I will earn more love*, *care*, *and respect from my family than what I am getting right now*.*” Participant 9*

*“My husband prefers baby boy*. *He says that if we will have a son*, *he will earn money and take care of us*, *but daughter will go to others house and she cannot give us anything*. *“Participant 3*

On contrary some participants also reported that, despite there was family pressure for a son, they do not care about that and are happy with whatever the outcome would be. But they are worried that if a girl baby is born then she might have to be pregnant again to fulfill family wish.

*“For me it is okay whether it will be a son or a daughter*, *but my husband believes son is needed in a family to carry out the family legacy*. *If I will not have a son*, *then I have to become pregnant again*.*” Participant 1*

#### Inadequate family care and support

Participants felt that they were not getting extra care and support from their family, particularly from their husband. Before the pregnancy, they thought their pregnancy would bring them more love and respect from others, but they did not find so. On the other hand, for some who were also student had difficulty in balancing their study with household chores. One young participant shared her experience on this;

*“When I knew that I was pregnant then I expected that family will be more caring and loving*, *they would help me visit the health facility*, *allow me to take rest and offer me proper food but none of these thing happened*.*” Participant 10*

One participant shared on how her husband’s negligence was upsetting her;

*“My husband has no time for me and is always busy with his work*. *He has never accompanied me to the health institution*. *I miss him during the checkups” Participant 3*

#### Disturbed family environment

Family environment was revealed to be an important concern for most of the participants. During pregnancy, they had to spend most of the time in family but troubled relationship among the family members created a disturbed ambience in the family. Another factor explored for their worry was *financial hardship* due to limited income and bigger family size. Moreover, such disturbed environment prevented them from asking any favor or sharing any difficulties with family members. One of the participants shared her experience on this;

*“In my family*, *I hardly find any peace*, *there is always quarrel between my husband and his brother*. *I feel so wretched due to this*. *I never share my disturbed feeling or problem with anyone because if I share they will be worried*. *So*, *the best way is to be quiet*.*” Participant 11*

Another participant reported:

*“After becoming pregnant*, *I found that our taste changes and we desire for different nutritious food*, *but it is difficult to manage in a bigger family*.*” Participant 4*

## Discussion

The aim of the study was to identify the risk factors associated with antepartum depressive symptoms. To the best of our knowledge, this is the first mixed method study on antepartum depressive symptoms using EPDS tool in public health facilities of Nepal. Although the study was limited to women who sought antenatal checkup, a high majority (83.6%) of women had received at least one ANC check-up in Nepal [26].

The study reported 18% of the pregnant women with depressive symptoms who visited public health facility for ANC check-up. Multivariable analysis showed four of the factors assessed were significantly associated at 95% CI namely; gestational period, health problem, alcohol intake by partner and sex preference of the child. The qualitative study further revealed that women with depressive symptoms were apprehensive on; birth outcome, a family expectation of male child, inadequate support from the family/husband and disturbed family environment.

The proportion of pregnant women with depressive-symptoms was relatively similar to other studies from South Asia: both, Bangladesh[14] and Pakistan, [27]reported 18% and India reported 14.4% [28]. Whereas, other studies reported higher prevalence; a review study of 51 different studies in LMACs reported 25.3% pooled prevalence [29], and in Ethiopia using the EPDS tool, depression was reported among 24.9% of the women attending antenatal clinic [24]. In Eastern Nepal, a hospital-based study carried out using Hamilton-Depression Scale, reported that half of the pregnant women had some form of depression [11]. Likewise, a recently published study in the earthquake-affected district of Nepal using EPDS tool reported 39% of pregnant women with common mental disorder[12]. This reporting could be higher because of the traumatic setting in the earthquake-affected district.

Though there is a difference in the extent to which depression is reported due to methodological variation like types of screening tool used, gestational time point when screened for symptoms, sample size and sample characteristics. Nevertheless, all the studies have shown the notable risk of depression during the pregnancy.

Similar to this study, other studies also reported the association of antenatal depression with gestational age[30], preference for a male child [31] and alcohol intake by the partner [9,32]. Several studies have assessed antepartum depression in the later stage of pregnancy [14,15,28]. On the other hand some studies have included all stages of pregnancy and reported prevalence of antepartum depression varied by gestational period [24,30] similar to this study. Such changes could be due to changes in physiology and social role.

This study found higher odds of depression in women whose spouse consumed alcohol. It is evident that spousal alcohol intake is associated with intimate partner violence [33–36]. Furthermore, the alarming prevalence (~28%) of spousal violence in Nepal [37] stresses a need for further studies to explore its association with antepartum depression. Another factor that strongly revealed from the study was sex preference, particularly for the male child, which is very common in Nepal due to ingrained social belief for lineage perpetuation [38,39]. Hence, as revealed from qualitative finding, Nepalese women are under immense family and social pressure to have a male child [40]. Besides, lack of household care and support makes pregnant women vulnerable to loss of motivation and self-care [41] and is a risk factor for antepartum depression[14,31]. In this study, women reported to agonize over lack of support for health care check-up, burdened household chores and inadequate love and care from family including the husband. During pregnancy, women mostly expects care and support from their husband, and unmet expectation distressed them.

Women’s past experiences of spontaneous or elective abortion in previous pregnancy caused anxiety and depression in subsequent pregnancy[31,42–44]. In univariate analysis, the factor was significantly associated with the antepartum depression and qualitative study also explored further onto it. The intrusive thoughts regarding the similar outcome for the present pregnancy might result in a significant cognitive and behavioral effect. Some of the women who had abortions previously that we interviewed shared similar thoughts. Another factor, gravidity was significantly associated with antepartum depression in univariate analysis. Type of experience in previous pregnancy whether positive or negative could influence their mental state in current pregnancy, and association with antepartum depression has been reported in other studies as well [41,42]. Unplanned pregnancy has also been reported a significant factor for antepartum depression [24,30,31], however, our study did not show any significant association.

During ANC check-ups, health workers routinely gather information on obstetric history, including elective/spontaneous abortions, but they seldom further explore the impact of such obstetric history on pregnant women. The antenatal check-up is also a good opportunity for health care providers to assess the depression and counsel. This is also an opportunity where health worker can involve the husband or other family member in counseling. In resource constraint setting, like Nepal where there are no specialist mental health workers at community-level, trained community health workers might be the feasible option in delivering mental health services during pregnancy [45,46].

### Limitation

The study had smaller sample size of pregnant women and gathered information only from public health facilities. Thus, findings from this study might not be generalizable to all the pregnant women in the community. Given the cross-sectional nature of the study, causal inference cannot be made between antepartum depressive symptoms and its risk factors. A further limitation of the study is that the EPDS tool has not been validated with gold standard tool to assess antepartum depression in Nepal. As the interviews were conducted after ANC check-up, interaction of women with health workers might have influenced their psychological state and response. The other limitation is that key variables like history of depression or co-morbid existence of anxiety were not assessed. Moreover, only 12 of total women, identified with antepartum depressive symptoms, consented for further in-depth interviews. Thus, if we were able to enroll all of them or could have used saturation theory, we could have better understanding about the risk factors.

## Conclusion

Notable proportion (18%) of women accessing ANC service were identified with depressive symptoms that stresses a need to integrate psychological care during pregnancy into the primary health care service. Preference for male baby was found to be a key risk factor for the depressive symptoms that was revealed from both quantitative and qualitative method. The study strongly highlights a need for further strengthening of ongoing efforts to minimize gap in gender equity at utmost.

## Supporting information

S1 File This is dataset. (SAV)

S2 File This is original questionnaire. (PDF)

S3 File This is English questionnaire. (PDF)
