# Supplementary material for: Understanding the antepartum depressive symptoms and its risk factors among the pregnant women visiting public health facilities of Nepal
Source: PLoS One. 2019 Apr 4;14(4):e0214992. doi: 10.1371/journal.pone.0214992 (PMC6448918; doi:10.1371/journal.pone.0214992)
Supplement: S2 File — (PDF) [file pone.0214992.s002.pdf]

कोड नं-......

सि.नं.	प्रश्नहरु	सांकेतिक/नियमावली श्रेणीहरु	छाड्ने
**भाग १: सामाजिक सामाजिक- जनसंखिस बिशेषताहरु/कारणहरु**			
१०१	तपाई कति बर्षको हुनुभयो?	☐☐	
१०२	बिवाह गर्दा तपाईको उमेर कति थियो?	☐☐	
१०३	बिवाहको प्रकार		
१०४	तपाईको बिवाह भएको कति बर्ष भयो?		
१०५	पहिलो पटक गर्भवती हुदा कति बर्षको हुनुहुन्थ्यो?	☐☐	
१०६	अहिले तपाईको कति जना बच्चा घन्टा सुत्सनु छन ?	केटा ☐ केटी ☐	
१०७	तपाईको बच्चाको उमेर कति भयो?	१) ..... २) ...... ३)	
१०८	तपाईको परिवारको किसिम के हो?	एकल.......................१<br>संयुत..................... २<br>बृहत.........................३	
१०९	परिवारमा कति जना सदस्य हुनुहुन्छ?	☐☐	
११०	तपाई कहाँ बस्नुहुन्छ?	आफ्नै घर ....१<br>भाडामा ....२<br>अन्य ३	
१११	तपाईले कति पढ्नुभएको छ?	निरक्षर......................१<br>साक्षर........................२<br>प्राथमिक……………....३<br>निम्नमाध्यमिक...........४<br>माध्यमिक..................५<br>उच्चमाध्यमिक............६<br>स्नातक.......................७<br>स्नातकोत्तर..................८	
११२	तपाईको पेसा के हो?		
११३	जाति	ब्राहामण.....................१<br>क्षत्री.........................२<br>जनजाति......................३<br>दलित..........................४<br>अन्य____________८ (खुलाउने)	
११४	धर्म	हिन्दू ....१<br>बौद्ध...............................२	

		मुस्लिम..........................३<br>क्रिस्चियन........................४<br>अन्य_____________८ (खुलाउने)	
११५	आम्दानिको मुख्य स्रोत	कृषि..........................१<br>ब्यापार.......................२<br>नोकरी........................३<br>मजदूरी......................४<br>बैदेशिक रोजगारी.........५<br>अन्य_____________८ (खुलाउने)	
११६	श्रीमानको शिक्षा अवस्था	निरक्षर.....................१<br>साक्षर.......................२<br>प्राथमिक……………....३<br>निम्नमाध्यमिक..........४<br>माध्यमिक.................५<br>उच्चमाध्यमिक............६<br>स्नातक.......................७<br>स्नातकोत्तर..................८	
११७	तपाईको श्रीमानको पेसा के हो?		
**भाग २: गर्भअवस्था सम्बन्धी**			
२०१	गर्भअवस्थाको महिना?	[ ]	
२०२	तपाईले कति पटक गर्भअवस्थाको जाँच गाराउनुभयो?	[ ][ ]	
२०३	तपाईलाई गर्भवती हुनुभन्दा पहिलेदेखी नै कुनै स्वास्थ्य समस्या थियो ?	थियो ....१<br>थिएन २	→ २०५
२०४	कस्तो किसिमको स्वास्थ्य समस्या थियो ?	…………………………(खुलाउने)	
२०५	तपाईलाई अहिले कुनै स्वास्थ्य समस्या छ?	छ ....१<br>छैन २	→ २०८
२०६	कुन/कस्तो स्वास्थ्य समस्या छ ?	....................................(खुलाउने)	
२०७	यो समस्याको लागि तपाईले उपचार गर्नुभयो ?	छ ....१<br>छैन २	
२०८	तपाईलाई गर्भअवस्था सम्बन्धी कुनै स्वास्थ्य समस्या छ?	छ ....१<br>छैन २	
२०९	गर्भअवस्था सम्बन्धी कस्तो स्वास्थ्य समस्या छ?	.... (खुलाउने)	
२१०	के तपाईले यस गर्भअवस्थाको लागि योजना बनाउनुभएको ?	थियो ....१<br>थिएन २	→२१२
२११	तपाईको यो गर्भ चाहेको गर्भ हो?	हो ....१<br>होइना २	

२१२	पाहिले तपाईको गर्भ खेर(तुहिएको) गएको थियो?	थियो ....१<br>थिएन २	→ २१४
२१३	कति पटक?	१ पटक ....१<br>२ पटक ....२<br>३ पटक ....३<br>३ पटक भन्दा बढी ४	
२१४	के तपाईले कहिल्यै गर्भ पतन गराउनुभएकोछ ?	छ ....१<br>छैन २	→ २१५
२१५	कति पटक गर्भपतन गराउनुभयो?		
**भाग ३: परिवार सम्बन्धी**			
३०१	तपाई अहिले धुम्रपान गर्नुहुन्छ?	गर्छु.................................१<br>गर्दिन २	→ ३०३
३०२	तपाई कति धुम्रपान गर्नुहुन्छ?	कहिले काही ....१<br>प्रायजसो २	
३०३	तपाई अहिले मध्यपान गर्नुहुन्छ?	गर्छु.................................१<br>गर्दिन २	→ ३०५
३०४	तपाई कति मध्यपान गर्नुहुन्छ?	कहेले काही ....१<br>प्रायजसो २	
३०५	तपाईको श्रीमानको धुम्रपान गर्ने बानी छ ?	गर्छ ....१<br>गर्दैन २	→ ३०७
३०६	कति धुम्रपान गर्नुहुन्छ ?	कहेले काही ....१<br>प्रायजसो २	
३०७	तपाईको श्रीमानले मध्यपान गर्नुहुन्छ?	गर्छ ....१<br>गर्दैन २	→ ३०९
३०८	कति मध्यपान गर्नुहुन्छ?	कहेले काही ....१<br>प्रायजसो २	
३०९	तपाई कति घन्टा सुत्नु हुन्छ?	४ घंटा भन्दा कम................१<br>४ देखि ६ घंटा ....२<br>७ देखि ८ घंटा ....३<br>८ घंटा भन्ता भन्दा माथि ४	
३१०	के तपाई दिउँसोको समयमा आराम गर्नुहुन्छ?	गर्छु.................................१<br>गर्दिन २	
३११	के तपाई आफ्नो बैवाहिक जींवनबाट संतुष्ट हुनुहुन्छ?	छ ....१<br>छैन २	
३१२	के तपाईले बिगतको बर्षमा तनाबपूर्ण क्षण भोग्नुभको थियो ?	थियो ....१<br>थिएन २	

३१३	के तापाईको श्रीमान यस गर्भावस्थामा संगै हुनुहुन्छ?	छ ....१<br>छैन २	
**भाग ४: सांस्क्रितिक कारण सम्बन्धी**			
४०१	तपाई यो गर्भबाट के चाहानुहुन्छ?	छोरा ....१<br>छोरी ....२<br>जुनभए पनि हुन्छ ....३<br>थाहाछैन ४	→ ४०३<br>→ ४०३
४०२	तपाई किन छोरा/ छोरी हुनुपर्छ भन्ने सोच्नुहुन्छ?		
४०३	तपाईको श्रीमान / परिवारको छोरा / छोरी हुनुपर्छ भन्ने चाहना छ ?	छ ....१<br>छैन २	→ ४०५
४०४	छोरा अथवा छोरी के चाहानुहुन्छ?	छोरा ....१<br>छोरी २	
४०५	के तपाई परिवारको यो चाहनाबाट चिन्तित हुनुहुन्छ?	छ ....१<br>छैन २	
४०४	तपाईको श्रीमानको स्वभाव कस्तो छ?	नियन्त्रित ....१<br>लचिलो २	

EDINBURGH DEPRESSION SCALE

तपाई गर्भवती भएको हुंदा हामी तपाई कस्तो महसूस गरिराहनुभाको छ भन्ने जान्न चाहन्छौ कृपया तपाईले आज महसुस गर्नु भएको कुरा मात्र नभएर बिगत ७ दिन भित्रमा कस्तो महसुस गरिरहनु भएको छ आफ्नो भावनासंग मेल खाने उत्तर भन्नुहोस/छान्नुहोस I

१) म हांस्न सकेकी छु र हरेक कुराको रमाइलो पक्ष हेर्न सकेकी छु ।

- जति म सधैं गर्दै आएकी छु ।
- निश्चय पनि पहिले भन्दा कम ।
- अब त्यती सकिदैन ।
- बिल्कुल सकिदैन ।

२) म आउने कुरा हरु प्रती खुशी महशुस गर्छु ।

- पाहिले जति नै ।
- पाहिले भन्दा केही कम मात्र ।
- निश्चितरूप मा पहिले भन्दा कम ।
- पाहिले भन्दा एकदमै/असाध्यै कम ।

***३) कुनै पनि चीज बिग्रनुमा म अनाव्श्यकरूपमा आफुलाई दोषी ठान्ने गर्दछु ।**

- हो, अधिकांश समय ।
- हो, तर कुनै कुनै बेला मात्र
- प्रायजसो **मान्छु** ।
- कहिले पनि मान्दिन ।

४) पर्याप्त कारण नहुँदा पनि म डर र चिन्ता महशुस गरिरहन्छु I

- होइन् , बिल्कुल होइन ।
- एकदमै विरलै हुन्छ ।

***६) कुनै पनि दिनचर्याको काम मलाई बोझको रुपमा लाग्ने गर्दछ्न ।**

- हो, अधिकांश समय बोझ उठाउन सकिरहेकी छैन ।
- हो, कहिले काहिं पहिलेको जसरी बोझ उठाउन सकिरहेकी छैन ।
- होइन्, अधिकांश समय म राम्रो सँग सहन सकिरहेकी छु ।
- होइन्, म सधैं जसरी नै बोझ सहन सकिरहेकी छु ।

***७) म यती धेरै उदास महसुस गरिरहेकी छु कि मलाई सजिलै सँग निन्द्रा लागिरहेको छैन ।**

हो , अधिकांश समय।

- हो , कहिले काही ।
- खासै होइन।
- होइन

***८) मेरो मन उदास वा खिन्न रहन्छ ।**

- रहन्छ , अधिकांश समय ।
- रहन्छ , कहिलेकाहिं ।
- प्राय जसो रहन्छ ।
- कहिले पनि रहदैन ।

***९) म दु:खी भईरहेकी हुन्छु र रोइरहेकी हुन्छु ।**

- कहिले काही हुन्छ ।
- प्रायजसो हुन्छ ।

***५) पर्याप्त कारण नभए पनि तर्सिने वा अत्याधिक भयभित महसुस गर्नेगर्छु ।**

- हो, धेरै नै ।
- कहिले काही ।
- **होइन्, धेरै होइन ।**
- **होइन्, कहिले पनि होइन ।**

- हो, अधिकांश समय ।
- हो, प्रायजसो ।
  - कहिले कहिं मात्र ।
  - रहदिंन , कहिले पनि रहदिंन ।

***१०) आफैलाई चोट पुर्याउने सोच मलाई आएको छ:**

- हो, प्रायजसो ।
  - कहिले काही ।
  - होइन कमै त्यस्तो सोच आऊछ ।
  - कहिले पनि त्यस्तो सोच आएको छैन ।

कुल प्राप्तांक: _____________

SCORING

- प्रश्न १, २, र ४ (* नभएको)अंक ०,१,२, वा ३ पहिलोलाई ० र तलको(अन्तिम)लाई ३
- प्रश्न ३,५ - १० (* भएको) उल्टो अंक,पहिलोलाई ३ र तलको(अन्तिम)लाई ०
- सब भन्दा धेरै अंक: ३०,सम्भाबिद डिप्रेसन: १० वा १० भन्दा बढी

यदी उत्तरदाताले १० वा सो भन्दा कम ल्याए धन्यवाद दिनुहोस । यदी उत्तरदाताले १० वा सो भन्दा बढी ल्याए निम्न अन्तरबार्ता प्रश्नावलीमा जानुहोस ।

अन्तरबार्ता प्रश्नावली

Q1. कृपया भनिदिनुहोस, गर्भवती भएदेखी समग्रमा तपाईंको अनुभव कस्तो रह्यो ?

Q2. तपाईंलाई पहिलो पटक गर्भवती भएको थाहा पाउंदा कस्तो महसुस भएको थियो ? खोतल्नुहोस्: तपाईं हर्षित, दुखी, वा अचम्भित के हुनुभएको थियो ?

Q3. तपाईंले उक्त कुरा क-कसलाई भन्नुभयो ? खोतल्नुहोस्: परिवर/ श्रीमान/ साथीहरु को प्रतिकृया ।
Q4. तपाइ अहिले कस्तो महसुस गरिरहनुभएको छ ? किन भनेर खोतल्नुहोस ।

Q5. गर्भवती भएको थाहापाए पछी महिलाहरुले आफ्नो गर्भावस्था बाट विभिन्न अपेक्षा गरेका हुन्छन । तपाइको आफ्ना अपेक्षाहरु भन्नसक्नुहुन्छ ?

Q6. तपाइलाई के लाग्छ, के यो गर्भावस्थाले तपाईंको जिवनमा कुनै परिवर्तन ल्याउन सक्छ ?

Q7. तपाइ गर्भवती हुनुभएपछी तपाइको जिवनमा के कस्ता समस्याहरु देखिएका छन ? श्वास्थ्य, शिक्षा, कार्य तथा दैनिक जिवनमा । खोतल्नुहोस ।

Q8. के कस्ता कुराहरुमा तपाइ सुधार होस भन्ने चाहनुहुन्छ जसले तपाइलाई पहिले भन्दा बढी खुशी दिन सक्छ ?

अब हामी अन्तरवार्ताको अन्त्यमा आइसकेका छौ, अन्तरवार्ता सकिनुभन्दा अरु केहि थप भन्न चाहनुहुन्छ? या तपाइसँग अरु कुनै प्रश्न हरु छ्न्, जुन मलाई सोध्न चाहनुहुन्छ ?
