# Supplementary material for: Understanding the antepartum depressive symptoms and its risk factors among the pregnant women visiting public health facilities of Nepal
Source: PLoS One. 2019 Apr 4;14(4):e0214992. doi: 10.1371/journal.pone.0214992 (PMC6448918; doi:10.1371/journal.pone.0214992)
Supplement: S3 File — (PDF) [file pone.0214992.s003.pdf]

Code no-……

S.N	Questions	Coding Categories	Skip
**SECTION1: Socio-demographic characteristics/factors**			
101	How old were you in your last birthday?	☐☐	
102	How old were you when you get married?	☐☐	
103	Type of marriage		
104	Duration of marriage		
105	How old were you when you firstly got pregnant?	☐☐	
106	How many living children do you have?<br>If yes, how many boys and how many girls?	Boy ☐ Girl ☐	
107	Age of previous children		
108	What is the type of your family?	Nuclear…………..….1<br>Joint……………..…..2<br>Extended….................3	
109	How many members are in your family?	☐☐	
110	The house you live in, is it rented or your own or other?	Own house…………1<br>Rent…………………2<br>Other………………..3	
111	Could you please tell me about your educational status?	Illiterate ……..……...1<br>Literate………………2<br>Primary……………....3<br>lower Secondary…......4<br>Secondary…………....5<br>Intermediate……….....6<br>Bachelor……………...7<br>Master………………...8	
112	Could you please tell me about your occupational status?	Specify………………………	
113	Which ethnicity do you belong?	Brahmin……………....1<br>Chettri………………...2<br>Janjati……………….....3<br>Dalit ………………......4<br>Others____________8<br>(Specify)	
114	Which religion do you follow?	Hindu……………….....1<br>Buddhist……………....2<br>Muslim ……………..…3<br>Christian……………..…..4	

		Others______________8<br>(Specify)	
115	What is the main source of your household income?	Agriculture……………1<br>Business……………....2<br>Service………….......3<br>Labor…………………..4<br>Abroad…………………5<br>Other_______________8<br>(Specify)	
116	Could you please tell me about the educational status of your husband?	Illiterate ……..……....1<br>Literate………………2<br>Primary……………....3<br>lower Secondary…......4<br>Secondary………….....5<br>Intermediate……….....6<br>Bachelor……………...7<br>Master………………...8	
117	Could you please tell me about the occupational status of your husband?	Specify…………………….	
**SECTION2: Pregnancy Related Variables**			
201	Month of pregnancy?	☐	
202	How many times had you visited for ANC for this pregnancy?	☐☐	
203	Before this pregnancy did you suffer from any health problem?	Yes……………………..1<br>No………...……………2	→ 206
204	What types of health problem did you suffer?	…………………………<br>(Specify)	
205	Did you suffer from any health problem since (other than pregnancy related) pregnancy?	Yes…………………….1<br>No……………………..2	
206	Which health problem/complication do you have?	(Specify)	
207	Did you receive treatment for this?	Yes…………………….1<br>No……………………..2	
208	Do you have any health problem related to pregnancy?	Yes……………………..1<br>No………...……………2	→ 209
209	Which health problem/complication do you have, related to pregnancy?	…………………………<br>(Specify)	
210	Had you planned for this pregnancy?	Yes……………………..1<br>No………...……………2	→ 211
211	Is this your wanted pregnancies?	Yes……………………..1<br>No………...……………2	
212	Did you have miscarriage in previous time?	Yes……………………..1<br>No………...……………2	→ 213
213	How many times?	1 time..............................1	

		2 times............................2<br>3 times............................3<br>More than 3 times............4	
214	Have you ever done abortion?	Yes……………………..1<br>No………..……………2	
215	How many abortions have you done?	☐☐	
**SECTION3: Family Related Variables**			
301	Do you currently smoke cigarette?	Yes……………………..1<br>No………..……………2	→ 303
302	If yes, how often do you smoke?	Sometimes…………1<br>Often ………….……2	
303	Do you drink alcohol?	Yes……………………..1<br>No………..……………2	→ 305
304	If yes, how often do you drink?	Sometimes…………1<br>Often ………………2	
305	Does your husband have smoking habit?	Yes……………………..1<br>No………..……………2	→ 307
306	If yes, how often does he smoke?	Sometimes…………1<br>Often ………………2	
307	Does your husband have alcohol drinking habit?	Yes……………………..1<br>No………..……………2	→ 309
308	If yes, how often does he drink?	Sometimes…………1<br>Often ………………2	
309	How many hours do you sleep?	Below 4 hours …………1<br>4 to 6 hours…………….2<br>7 to 8 hours…………….3<br>Above 8 hours…………4	
310	Do you take rest in day time?	Yes……………………..1<br>No………..……………2	
311	Are you satisfied with your marital life?	Yes……………………..1<br>No………..……………2	
312	Did you face any stressful life events in previous year?	Yes……………………..1<br>No………..……………2	
313	Currently are you accompanied by your husband?	Yes……………………..1<br>No………..……………2	
**SECTION4: Cultural Related Factors**			
401	What do you expect from this pregnancy, a any or boy or girl?	Boy ....1<br>Girl ....2<br>Any ....3<br>Don’t know...................4	403
402	Why do think you want to have boy/girl?	…………………………..	
403	Is there such preference in your family/husband?	Yes……………………..1<br>No………..……………2	

404	What is the preference by your family/husband?	Boy ....1<br>Girl ....2<br>Any ....3<br>Don’t know...................4	
405	Are you worried of their expectation from this pregnancy?	Yes……………………..1<br>No………..……………2	
406	What’s type of behavior of your husband’s with you?	Controlling……………1<br>Flexible………………...2	

EDINBURG POSTNATAL DEPRESSION SCALE (EPDS)

We would like to know how you are feeling. Please mention which comes closest to how you have felt in the past 7 days, not just how you feel today.

1. I have been able to laugh and see the funny side of things as much as I always could.<br>o As much as I always could<br>o Not quite so much now.<br>o Definitely not so much now<br>o Not at all	2. I have looked forward with enjoyment to things.<br>o As much as I ever did<br>o Rather less than I used to<br>o Definitely less than I used to<br>o Hardly at all
3. I have blamed myself unnecessarily when things went wrong.<br>o Yes, most of the time.<br>o Yes, some of the time<br>o Not very often<br>o No, never	4. I have been anxious or worried for no good reasons.<br>o No, not at all.<br>o Hardly, ever<br>o Yes, sometimes<br>o Yes, very often
5. I have felt scared or panicky for no very good reason.<br>o Yes, quite a lot<br>o Yes, sometimes<br>o No, not much<br>o No, not at all	6. Things have been getting on top of me:<br>o Yes, most of the time I haven’t been able to cope at all<br>o Yes, sometimes I haven’t been coping as well as usual<br>o No, most of the time I have copied quite well<br>o No, I have been coping as well as ever
7. I have been so unhappy that I have had difficulty sleeping<br>o Yes, most of the time<br>o Yes, sometimes<br>o Not very often<br>o No, not at all	8. I have felt sad or miserable<br>o Yes, most of the time<br>o Yes, quite often<br>o Not very often<br>o No, not at all
9. I have been so unhappy that I have been crying<br>o Yes, most of the time<br>o Yes, quite often<br>o Only occasionally<br>o No, not at all	10. The thought of harming myself has occurred to me.<br>o Yes, quite often<br>o Sometimes<br>o Hardly ever<br>o Never

Questions 3,5,6,7,8,9 and 10 are scored: statement 1 = 3 points, statement 2 = 2 points, statement 3 = 1 point and statement 4 = 0 points. A cut-off score of 10 has been shown to detect minor and major depression and a woman with this threshold will be approached for further interview with guideline below. If less than 10, thank the respondent and end the interview.

Interview Guidelines

Thank you for making a time to participate in this study. Please do not hesitate to give answer and to tell what you feel or think. I will ensure that I won't disclose your identity for the information you will provide. The information you provide will cause no harm to you.

**Guiding questions**
Q1. Could you please tell me about your overall experience since becoming a pregnant?
Q2. When you first knew that you were pregnant how did you feel? Prompts: Were you Surprised or excited or worried?
Q3. Whom did you share this to? Prompts: Reaction of family/husband/friends
Q4. How are you feeling right now? Prompt 'why' for the response
Q5. A woman could have different expectations from her pregnancy after she knows about it. Could you tell me what yours was?
Q6. What do you think if this pregnancy will bring any changes in your live?
Q7. What problems have emerged in your life since you became pregnant? Prompts: health, work, education, daily life. Why?
Q8. What are the things you wish to improve so that you can become happier than now?
We are about to finish the interview, and before that I would like to ask is there is anything you would like to share on the matter we just discussed?<br>Do you have any questions that you would like to ask me?

Thank you for sharing your thoughts and experiences with me. It has been a great experience for me to interview you.

Take care, Thank you!
